# Balloon enteroscopy assisted endoscopic retrograde cholangiopancreatography with the rendezvous technique using a percutaneous transjejunal route

**DOI:** 10.1055/a-2808-7623

**Published:** 2026-03-24

**Authors:** Joji Muramatsu, Kazuma Ishikawa, Tomohiro Kubo, Kohichi Takada

**Affiliations:** 1 Division of Medical Oncology, Department of Internal Medicine, Sapporo Medical University School of Medicine, Hokkaido, Japan

**Fig. 1:**
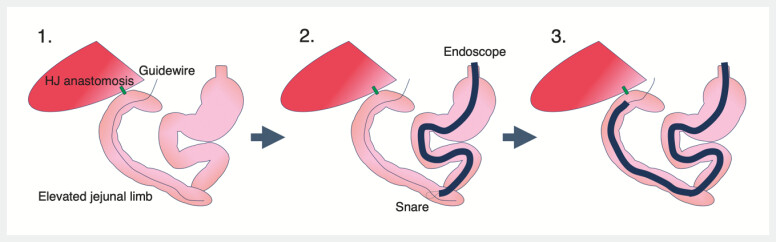
A schematic diagram of balloon enteroscopy assisted ERCP with the rendezvous technique using a percutaneous transjejunal route. First, an external fistula was percutaneously created at the blind end of the jejunal limb, and a guidewire was inserted through this route. Second, an endoscope was advanced, and the guidewire was grasped using a snare device. Finally, the endoscope was advanced into the hepaticojejunal anastomosis while traction on the guidewire was applied from the jejunostomy side. ERCP, endoscopic retrograde cholangiopancreatography.

Balloon enteroscopy assisted endoscopic retrograde cholangiopancreatography (BE-ERCP) is effective for biliary drainage after hepaticojejunostomy 1 2 ; however, access to the hepaticojejunal (HJ) anastomosis can be challenging due to long reconstructed intestinal tracts, severe angulation, or postoperative adhesions 3 . In such situations, endoscopic ultrasound guided biliary drainage (EUS-BD) or percutaneous transhepatic biliary drainage (PTBD) may be considered as alternative approaches. However, after left hepatectomy, EUS-BD is not feasible, and PTBD is difficult without bile duct dilation. We report a case in which the HJ anastomosis was difficult to reach and no alternative approaches were feasible, but access was achieved using a rendezvous technique via a percutaneous transjejunal route ( Fig. 1 and Video 1 ).

Successful balloon enteroscopy assisted ERCP using a percutaneous transjejunal rendezvous technique for difficult access to the hepaticojejunal anastomosis. ERCP, endoscopic retrograde cholangiopancreatography.Video 1

**Fig. 2:**
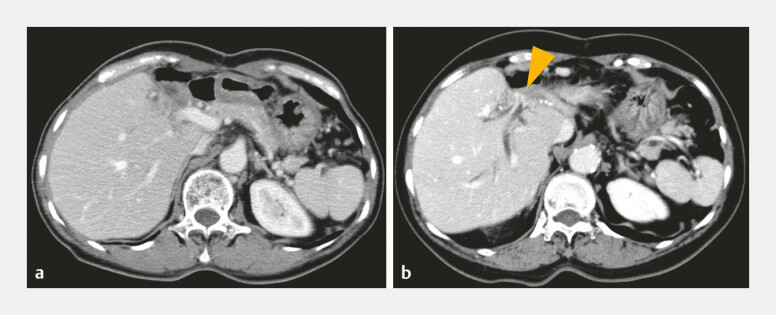
The benign stricture at the hepaticojejunal anastomosis. **a** X-21 year. **b** X-3 year. Orange arrowheads indicate the hepaticojejunal anastomotic stricture.

**Fig. 3:**
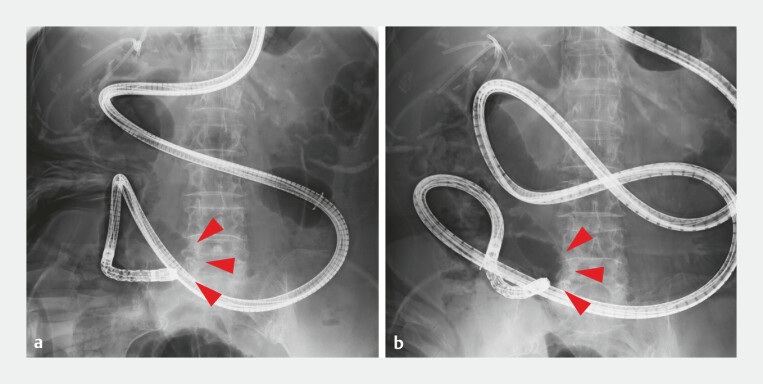
Difficulty in reaching the hepaticojejunal anastomosis using BE-ERCP. **a** X-1 year. **b** X-year. Red arrowheads indicate the site of severe angulation and jejunal adhesions. BE-ERCP, balloon enteroscopy assisted endoscopic retrograde cholangiopancreatography.

An 80-year-old woman who had undergone left hepatectomy and hepaticojejunostomy for echinococcosis developed a benign stricture at the HJ anastomosis ( Fig. 2 ). Over the preceding 3 years, recurrent biliary stone formation required multiple bile duct stone extraction and stent placement procedures using BE-ERCP. However, severe angulation, jejunal adhesions, and stretching of other jejunal loops made access to the HJ anastomosis increasingly difficult, eventually precluding endoscopic access ( Fig. 3 ).

**Fig. 4:**
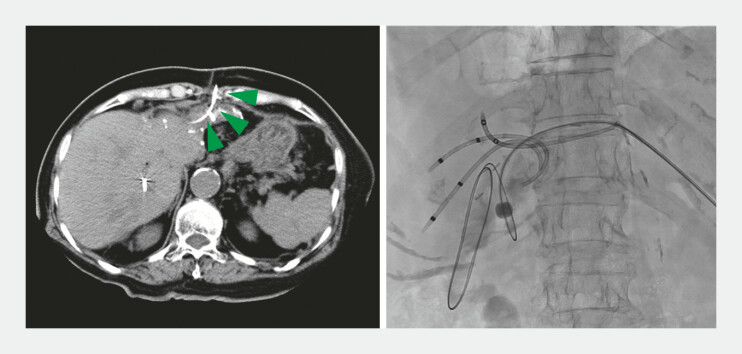
Percutaneous jejunostomy. Green arrowheads indicate the percutaneous jejunostomy route.

**Fig. 5:**
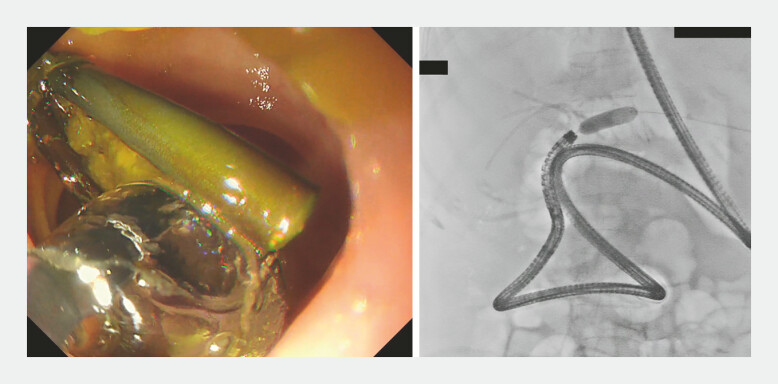
Reattempted BE-ERCP in X year. The scope was inserted by aligning its axis with the intestinal axis, using the traction of the guidewire. BE-ERCP, balloon enteroscopy assisted endoscopic retrograde cholangiopancreatography.

A percutaneous jejunostomy was therefore created ( Fig. 4 ). After sufficient maturation of the fistula, BE-ERCP was reattempted. A guidewire was inserted through the jejunostomy, allowing scope insertion by aligning the endoscope axis with the intestinal axis using guidewire traction ( Fig. 5 ). The dilating balloon was used to straighten the angulated intestinal axis rather than to dilate the stricture. The HJ anastomosis was reached, and biliary intervention was completed.

The external drainage tube in the permanent jejunostomy protruded only a few centimeters outside the skin and allowed repeated endoscopic procedures without compromising the quality of life. This is the first report demonstrating that a percutaneous transjejunal rendezvous approach combined with BE-ERCP can provide access to a difficult HJ anastomosis while avoiding surgical intervention and the complexities associated with PTBD.

Endoscopy_UCTN_Code_CCL_1AZ_2AZ Endoscopy_UCTN_Code_TTT_1AO_2AK Endoscopy_UCTN_Code_TTT_1AR_2AG
